# Energy requirements for critically ill patients with COVID‐19

**DOI:** 10.1002/ncp.10852

**Published:** 2022-03-21

**Authors:** Ryan Burslem, Kimberly Gottesman, Melanie Newkirk, Jane Ziegler

**Affiliations:** ^1^ Department of Clinical and Preventive Nutrition Sciences School of Health Professions Rutgers University Newark NJ USA; ^2^ Department of Clinical and Preventive Nutrition Sciences Rutgers University Newark NJ USA; ^3^ Department of Clinical and Preventive Nutrition Sciences Clinical Nutrition Program Rutgers University Newark NJ USA

**Keywords:** COVID‐19, critical illness, energy requirements, indirect calorimetry, nutrition support, SARS‐CoV‐2

## Abstract

Early reports suggested that predictive equations significantly underestimate the energy requirements of critically ill patients with coronavirus disease 2019 (COVID‐19) based on the results of indirect calorimetry (IC) measurements. IC is the gold standard for measuring energy expenditure in critically ill patients. However, IC is not available in many institutions. If predictive equations significantly underestimate energy requirements in severe COVID‐19, this increases the risk of underfeeding and malnutrition, which is associated with poorer clinical outcomes. As such, the purpose of this narrative review is to summarize and synthesize evidence comparing measured resting energy expenditure via IC with predicted resting energy expenditure determined via commonly used predictive equations in adult critically ill patients with COVID‐19. Five articles met the inclusion criteria for this review. Their results suggest that many critically ill patients with COVID‐19 are in a hypermetabolic state, which is underestimated by commonly used predictive equations in the intensive care unit (ICU) setting. In nonobese patients, energy expenditure appears to progressively increase over the course of ICU admission, peaking at week 3. The metabolic response pattern in patients with obesity is unclear because of conflicting findings. Based on limited evidence published thus far, the most accurate predictive equations appear to be the Penn State equations; however, they still had poor individual accuracy overall, which increases the risk of underfeeding or overfeeding and, as such, renders the equations an unsuitable alternative to IC.

## INTRODUCTION

Coronavirus disease 2019 (COVID‐19) was declared a pandemic by the World Health Organization (WHO) on March 11, 2020.^1^ As of early December 2021, there have been >260 million cases of COVID‐19 worldwide, with >5 million deaths.^2^ Most cases of COVID‐19 are asymptomatic or mild; however, ~14% of patients will develop severe illness prompting admission to a hospital and/or intensive care unit (ICU).^3^ Age is the strongest predictor of severe COVID‐19, specifically seen in adults aged >65 years.^4^ Additional at‐risk groups include those from minority populations and those with comorbidities, including diabetes, obesity, cancer, and chronic liver, lung, heart, and kidney disorders.^4^ Complications of severe COVID‐19 include acute respiratory distress syndrome (ARDS) and multiple organ failure.^5^ ARDS is thought to manifest both directly from severe acute respiratory syndrome coronavirus 2 (SARS‐CoV‐2) and indirectly from an overexaggerated immune response with excessive proinflammatory cytokine release, resulting in a cytokine storm.^5^ This cytokine storm, in turn, may lead to a hypometabolic or hypermetabolic state.

An early publication reported indirect calorimetry (IC) measurements from a small sample of critically ill patients with COVID‐19 and found that measured resting energy expenditure (mREE) was significantly higher than that predicted by the Penn State equation, a predictive equation validated for patients receiving mechanical ventilation.^6^ The authors concluded that critically ill patients with COVID‐19 have a significant hypermetabolic response to illness that may be related to COVID‐19's cytokine storm.^6^

IC is considered the gold standard to measure energy expenditure in critically ill patients.^7^ IC measures volume of oxygen consumption (VO_2_) and volume of carbon dioxide production (VCO_2_), which are input as variables in the abbreviated Weir equation to calculate energy expenditure.^7^ Unfortunately, IC is not available in many institutions because of barriers including the cost, training, staffing, and time required to conduct studies.^8^ As such, many practitioners rely on predictive equations to estimate energy expenditure. However, if predictive equations significantly underestimate energy requirements for critically ill patients with COVID‐19, then reliance on these equations increases the risk of underfeeding and resulting malnutrition. Malnutrition, in turn, is associated with poorer clinical outcomes, including longer length of ICU stay and increased risk for infection and hospital mortality.^9^

The purpose of this narrative review is to summarize and synthesize current evidence comparing mREE via IC with predicted resting energy expenditure (pREE) via commonly used predictive equations in adult critically ill patients with COVID‐19.

## SEARCH STRATEGY

Literature searches were conducted between October 2021 and December 2021 using the PubMed, CINAHL, and Scopus databases. Search terms included “critical illness,” “critically ill,” “intensive care unit,” “ICU,” “COVID‐19,” “SARS‐CoV‐2,” and “indirect calorimetry.” The author screened for studies that reported IC results in adult critically ill patients with COVID‐19. Studies were included if the results of IC were compared with a predictive equation or if the results of IC were reported in kilocalories per kilogram of body weight. If a study reported results in kilocalories per kilogram of body weight but did not compare these results with a predictive equation, then the results were compared with clinical practice guideline recommendations for estimating energy requirements for critically ill patients, including the American Society for Parenteral and Enteral Nutrition (ASPEN) and Society of Critical Care Medicine (SCCM) recommendation of 25–30 kcal/kg/day^10^ and the European Society for Parenteral and Enteral Nutrition (ESPEN) recommendation of 20–25 kcal/kg/day.^11^ Conference abstracts and review articles were excluded. Reference lists of articles were hand searched for additional relevant articles not captured by the initial database searches.

**Figure 1:**
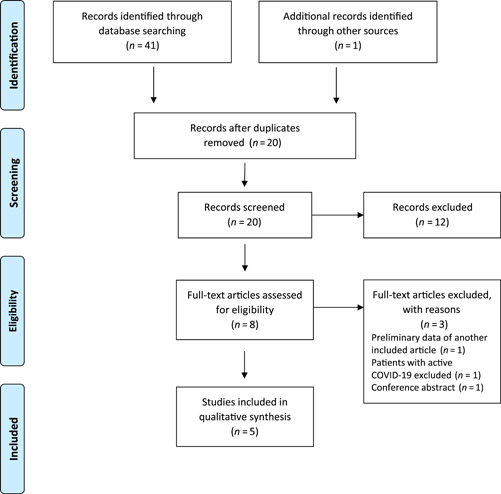
Preferred Reporting Items for Systematic Reviews and Meta‐Analysis 2009 flow diagram: selecting studies for narrative review. COVID‐19, coronavirus disease 2019

Figure 1 shows an overview of the search strategy and results. The database searches yielded a total of 41 records, with one additional article identified via manual review of reference lists. After removing duplicates, the remaining 20 articles were screened via review of titles and abstracts. Twelve of the articles were excluded in this manner; the remaining eight articles were reviewed in full for eligibility. Three articles were excluded after review: one article was a preliminary data set for another article included in this review; one article excluded patients with active COVID‐19; and one article was a conference abstract. The remaining five articles are summarized and synthesized below and in the table of related literature (Table 1). The quality of each study was graded as positive (+), neutral (Ø), or negative (‐), according to the Academy of Nutrition and Dietetics’ evidence analysis process.

**Table 1:** Studies which reported the results of IC in critically ill patients with COVID‐19

**Author, year, study design, country, funding source**	**Quality grade**	**Study purpose**	**Study population (demographics)**	**Intervention and setting**	**Outcome data**	**Conclusions/results**	**Limitations of findings**
Yu et al.,^6^ 2020, case series, United States, funding source: none	Neutral (Ø)	To report the results of IC measurements in critically ill patients with COVID‐19	7 critically ill adult patients with COVID‐19 receiving mechanical ventilation Age, years (median, range): 62 (55–74) Male: 5 (71.4%)	IC measurements taken and compared with pREE via Penn State equation	Median mREE = 4044 kcal/day (235.7% ± 51.7% of predicted) No strong correlation between mREE and serum inflammatory markers (C‐reactive protein and D‐dimer)	Critically ill patients with COVID‐19 are in a significant hypermetabolic state.	Limited details on how patients were selected/enrolled. REE not tracked over time. Lack of reporting on the following: Procedure for conducting IC Results in kcal/kg/day Specific Penn State equation used (2003b vs 2010)
Yu et al.,^12^ 2020, case series, United States, funding source: none	Neutral (Ø)	To report on hypermetabolism measured in four critically ill patients with COVID‐19 and to demonstrate how therapeutic hypothermia reduced metabolic demand	4 critically ill adult patients with COVID‐19 receiving mechanical ventilation Age, years (median, range): 66 (57–73) Male: 3 (75%) Obese: 2 (50%)	IC measurements taken and compared with pREE via Mifflin St Jeor equation. Therapeutic hypothermia induced for 48 h at a target temperature of 34.5°C; changes in mREE, VCO_2_, and VO_2_ recorded.	Case 1: mREE = 4282 kcal/day (278.8% of predicted) Case 2: mREE = 3728 kcal/day (282.6% of predicted) Case 3: mREE = 4381 kcal/day (261.7% of predicted) Case 4: mREE = 6490 kcal/day (374.7% of predicted) Median mREE = 4332 kcal/day Mean reductions in mREE, VCO_2_, and VO_2_ postcooling of 27.0%, 29.2%, and 25.7%, respectively	Critically ill patients with COVID‐19 are in an extreme hypermetabolic state. Therapeutic hypothermia reduced metabolic demand.	Limited details on how patients were selected/enrolled. REE not tracked over time. Lack of reporting on the following: Inclusion/exclusion criteria related to IC measurements Procedure for conducting IC Results in kcal/kg/day Days that IC measurements were taken post‐ICU admission Activity factor used (if any) for Mifflin St Jeor equation
Lakenman et al.,^13^ 2021, Prospective cohort study, the Netherlands, funding source: none	Positive (+)	To assess energy expenditure, gastrointestinal tolerance, and feeding practices during the acute and late phases of illness in critically ill patients with COVID‐19	21 critically ill adult patients with COVID‐19 Age, years (median, IQR): 59 (44–66) Male: 14 (66.7%) BMI: Normal: 5 (23.8%) Overweight: 4 (19.0%) Obese: 12 (57.1%) APACHE IV score, median (IQR): 22.2 (9.5–35.0)	IC measurements taken for each patient during acute phase (days 0–7) and late phase (day ≥8) of ICU admission. Results compared with pREE via WHO equation (BMI ≤ 30) or Harris‐Benedict equation (BMI > 30)	Mean mREE ± SD, acute vs late phase (kcal/day): 2267 ± 668 vs 2284 ± 623 (*P* = 0.529). Not significantly different, though mREE was higher in late phase for most patients. Hypometabolic (mREE <90% of pREE): 1 (5.0%) Normometabolic (mREE 90%–110% of pREE): 6 (30.0%) Hypermetabolic (mREE >110% of pREE): 13 (65.0%) mREE was significantly higher than pREE during acute (*P* = 0.001) and late (*P* = 0.000) phases	There were no differences in mREE between acute and late phases; however, most patients were hypermetabolic and subsequently underfed.	Acute and late phases defined based on admission date to study ICU, though most patients in study ICU were transferred from other ICUs, which may have introduced bias. Most late phase measurements were taken during the second week, limiting assessment of mREE after second week. Results not reported in kcal/kg/day.
Karayiannis et al.,^14^ 2021, prospective cohort study, Greece, funding source: none	Positive (+)	To measure energy expenditure in critically ill patients with COVID‐19 and to determine the impact of neuromuscular blockade on energy expenditure	34 critically ill adult patients with COVID‐19 on mechanical ventilation Age (years), mean ± SD: 65.9 ± 17.9 Male: 20 (58.8%) BMI: Overweight: 9 (26.4%) Obese: 12 (35.2%) APACHE II, median (IQR): 18.9 (11.0–26.2)	IC measurements taken on 3^rd^, 7^th^, 14^th^, 21^st^, and 28^th^ day of ICU admission	Median mREE on days 3, 14, 21, and 28: Nonobese: 17.8, 29.4, 31.1, and 29.3 kcal/kg/day, respectively, based on actual body weight (*P* = 0.011, day 3 vs day 28) Obese: 18.1, 27.2, 26.8, and 29.3 kcal/kg/day, respectively, based on adjusted body weight (*P* = 0.021, day 3 vs day 28) Median mREE in patients who received neuromuscular blockade for at least 3 days vs those who did not: 2444 vs 2120 kcal/day (*P* = 0.023)	Energy expenditure in critically ill patients with COVID‐19 increases significantly after the first week of ICU admission and stabilizes after the third week. Neuromuscular blockade significantly decreases energy expenditure.	Unclear how long the hypermetabolic phase lasted after the 4‐week study period Unclear variance in individual IC measurements Steady state parameters not fully defined
Niederer et al.,^15^ 2021, prospective cohort study, United States, funding source: Baxter	Positive (+)	To assess trends over time in mREE in critically ill patients with COVID‐19 and to compare mREE with several commonly used predictive equations	38 critically ill adult patients with COVID‐19 receiving mechanical ventilation Age, years (median, range): 61 (25–88) Male: 23 (61%) Race: Black: 18 (47%) White: 7 (18%) Other: 13 (34%) Ethnicity: Hispanic: 11 (29%) BMI (median): 31.8 ± 1.4 Obese: 22 (58%)	IC measurements taken over the course of 7 weeks post‐ICU admission and compared with pREE via Harris‐Benedict, Mifflin St Jeor, Penn State (2003b and 2010), and ASPEN/SCCM guideline recommendation equations	Mean mREE, overall (kcal/kg/day ± SEM): Week 1: 21.6 ± 1.1 Week 2: 23.1 ± 2.4 Week 3: 28.0 ± 1.9 Weeks 4–7: 27.9 ± 2.1 Mean mREE, nonobese patients vs those with obesity (kcal/kg/day ± SEM): Week 1: Nonobese: 25.1 ± 1.8 Obese: 19.5 ± 1.0 (*P* < 0.01) Weeks 2–3: Nonobese: 28.0 ± 2.0 Obese: 19.5 ± 1.5 (*P* < 0.01) Harris–Benedict, Mifflin St Jeor, and ASPEN/SCCM lower end of range underestimated mREE. Penn State and ASPEN/SCCM higher end of range were most accurate on average, though frequently underestimated/overestimated mREE in individual measurements.	Critically ill patients with COVID‐19 exhibit a progressive, prolonged hypermetabolic response to illness, which peaked by week 3 and was more pronounced in nonobese patients. Most predictive equations underestimated mREE. Penn State and ASPEN/SCCM upper end were most accurate.	mREE of patients with obesity compared with ASPEN/SCCM hypocaloric/high‐protein feeding guidelines, limiting interpretation of the accuracy of these equations to estimate REE Unclear how long the hypermetabolic phase lasts beyond the 7‐week study period

*Note:* BMI is calculated as weight in kilograms divided by height in meters squared.

Abbreviations: APACHE II, Acute Physiology and Chronic Health Evaluation II; ASPEN, American Society for Parenteral and Enteral Nutrition; BMI, body mass index; COVID‐19, coronavirus disease 2019; IC, indirect calorimetry; ICU, intensive care unit; mREE, measured resting energy expenditure; pREE, predicted resting energy expenditure; SCCM, Society of Critical Care Medicine; VCO_2_, volume of carbon dioxide production; VO_2_, volume of oxygen consumption.

## LITERATURE REVIEW

All five studies were conducted in adult critically ill patients with COVID‐19 receiving mechanical ventilation, with the exception of Lakenman et al.,^13^ in which mechanical ventilation was not specifically an inclusion criteria.

In 2020, Yu et al.^6^ published a case series reporting IC measurements in seven patients. Five of these patients were male (71%).^6^ The authors included patients who had persistent hypercapnia and/or hypoxia despite ventilator optimization, though specific inclusion criteria regarding these parameters were not defined.^6^ Measurements were taken between hospital days 8 and 55.^6^ Median mREE was 4044 kcal/day, which was 235.7% ± 51.7% of that predicted by the Penn State equation.^6^ Median VO_2_ was 585 ml/min.^6^ There was no strong correlation between mREE and serum inflammatory markers, including C‐reactive protein and D‐dimer.^6^ The authors concluded that critically ill patients with COVID‐19 are in a significant hypermetabolic state.^6^ This study earned a neutral rating, with limitations including limited details on how patients were selected and enrolled; minimal description on the procedure for conducting IC, such as no description of how steady state parameters were defined, which could affect the reliability of the results; small sample size; the range of days in which IC was conducted (days 8–55); results not reported in kilocalories per kilogram per day, limiting the comparison with other studies; and uncertainty with which Penn State equation was used (2003b vs 2010).

In a separately published case series with a different sample population, Yu et al.^12^ reported the results of IC measurements in four patients. Three of these patients were male (75%).^12^ Patients were described as having ongoing hypercapnia and/or hypoxia despite ventilator optimization.^12^ mREE for these patients ranged from 3728 to 6490 kcal/day (median, 4332 kcal/day), or 261.7%–374.7% of that predicted by the Mifflin St Jeor equation.^12^ Therapeutic hypothermia attenuated the hypermetabolic response, with mean reductions in mREE, VCO_2_, and VO_2_ postcooling of 27.0%, 29.2%, and 25.7%, respectively.^12^ Again, the authors concluded that critically ill patients with COVID‐19 are in a significant hypermetabolic state.^12^ This study also earned a neutral rating, with similar limitations to the previous case series. In addition to those described above, these limitations include lack of reporting days post‐ICU admission that measurements were taken and lack of reporting inclusion/exclusion criteria related to conducting the IC measurements. Given that the patients were described as having refractory hypoxia, inaccurate results may be obtained, for instance, if a patient's fraction of inspired oxygen exceeded the manufacturer's guideline cutoffs.

In 2021, Lakenman et al.^13^ published the results of a prospective cohort study reporting IC measurements in 21 patients during the acute phase (days 0–7) and late phase (day >7) of illness. Measurements were taken for a minimum of 10 min, with a steady state defined as a covariance <10% for VO_2_ and VCO_2_.^13^ mREE was compared with the WHO equation for nonobese patients (body mass index [BMI] ≤ 30 [calculated as weight in kilograms divided by height in meters squared]) or with the Harris–Benedict equation for patients with obesity (BMI > 30), both adjusted with predefined stress factors: +0% to 10% if sedated/mechanically ventilated; +10% to 20% if not sedated/mechanically ventilated; and +20% to 30% if not mechanically ventilated.^13^ Mean mREE ± SD during the acute and late phases was 2267 ± 668 and 2284 ± 623 kcal/day, respectively.^13^ For the majority of patients, mREE was higher in the late phase than in the acute phase, though the mean difference was not statistically significant (*P* = 0.529).^13^ Of note, most of the late phase measurements were taken during the second week of ICU admission.^13^ Measured REE was significantly higher than pREE during both the acute (*P* = 0.001) and late (*P* = 0.000) phases.^13^ When classifying patients as hypometabolic (mREE < 90% of pREE), normometabolic, or hypermetabolic (mREE > 110% of pREE), the majority of patients were hypermetabolic (65%), 30% were normometabolic, and 5% were hypometabolic.^13^ The authors concluded that there was no difference in mREE between the acute and late phases, though most patients were hypermetabolic and subsequently underfed.^13^ This study earned a positive rating. One noteworthy limitation is that the authors defined the acute and late phases based on admission day to the study ICU^13^; given that most patients had transferred from other ICUs, defining phases in this manner may have introduced bias in the interpretation of differences between the acute and late phases. Additional limitations include lack of assessment of mREE after the second week of ICU admission and lack of reporting IC measurements in kilocarlories per kilogram per day.

Next, Karayiannis et al.^14^ conducted a prospective cohort study with a sample of 34 patients. IC measurements were taken on the 3rd, 7th, 14th, 21st, and 28th day of ICU admission (though day 7 measurements were not specifically reported).^14^ In nonobese patients, median mREE on days 3, 14, 21, and 28 was 17.8, 29.4, 31.1, and 29.3 kcal/kg/day, respectively, based on actual body weight; the difference in mREE between days 3 and 28 was found to be significant (*P* = 0.011).^14^ Similarly, for patients with obesity, median mREE on days 3, 14, 21, and 28 was 18.1, 27.2, 26.8, and 29.3 kcal/kg/day, respectively, based on adjusted body weight; again, the difference in mREE between days 3 and 28 was found to be significant (*P* = 0.021).^14^ Median REE was significantly lower in patients who received neuromuscular blockade for at least three days than in patients who did not (2444 vs 2120 kcal/day; *P* = 0.023).^14^ Overall, the authors found that energy expenditure progressively increased throughout ICU admission, peaking around week 3.^14^ Limitations with these findings include uncertainty as to how long the hypermetabolic phase persisted after the 4‐week study period and unclear variance in the individual IC measurements. Additionally, the authors noted that they only included steady state measures of at least 20 min, though the acceptable degree of covariance was not defined.^14^

Finally, Niederer et al.^15^ conducted a prospective cohort study assessing mREE in 38 critically ill patients up to 7 weeks post‐ICU admission. Measurements were taken for a minimum of 10 min, with a steady state defined as a covariance <10% for VO_2_ and VCO_2_.^15^ IC measurements were compared with the Harris‐Benedict, Mifflin St Jeor, Penn State (2003b and 2010), and ASPEN/SCCM lower‐end and upper‐end guideline recommendations.^15^ For nonobese patients, these recommendations were based on 25–30 kcal/kg/day; for patients with obesity, these recommendations were based on hypocaloric/high‐protein feeding guidelines.^15^ Mean mREE increased over the course of ICU admission, from 21.6 kcal/kg/day during week 1 to 27.9 kcal/kg/day during weeks 4–7.^15^ Measured REE was significantly higher in nonobese patients than in patients with obesity during week 1 (25.1 vs 19.5 kcal/kg/day; *P* < 0.01) and weeks 2–3 (28.0 vs 19.5 kcal/kg/day; *P* < 0.01).^15^ No comparisons were done between nonobese patients and those with obesity after week 3 because of low sample size.^15^ The Harris‐Benedict, Mifflin St Jeor, and ASPEN/SCCM lower end of range were found to consistently underestimate mREE.^15^ The Penn State equations and the ASPEN/SCCM upper end of range were found to be the most accurate overall, though these equations still frequently underestimated or overestimated mREE for individual patients; furthermore, 30 kcal/kg/day overestimated energy requirements during weeks 1–2.^15^ Overall, mREE was found to progressively increase over the course of ICU admission, peaking by week 3,^15^ similar to the findings of Karayiannis et al.^14^ However, in contrast to Karayiannis et al.,^14^ a progressive increase in mREE was not seen in patients with obesity.^15^ A significant limitation is the authors’ comparisons of mREE in patients with obesity with ASPEN/SCCM's guidelines for hypocaloric/high‐protein feeding, as these guidelines are not meant to estimate energy expenditure but rather to approximate meeting <70% of REE via IC; this limits interpretation of the accuracy of ASPEN/SCCM guideline recommendations to estimate REE. An additional limitation is uncertainty with how long the hypermetabolic phase lasts beyond the 7‐week study period.

## DISCUSSION

The hypermetabolism noted in the studies reviewed above may be explained by the heightened state of inflammation. Inflammation occurs as the body's natural response to resolve injury or infection.^16^ Inflammation is associated with the release of proinflammatory cytokines, such as tumor necrosis factor α (TNF‐α), interleukin 1 (IL‐1), and interleukin 6 (IL‐6), which aid in the recruitment of immune cells (such as neutrophils) to the site of injury or infection.^16^ These immune cells also release cytokines at the site of injury.^16^ In the case of critical illness, the inflammatory response becomes prolonged and exaggerated; ultimately, increasing numbers of proinflammatory cytokines may spill out into the systemic circulation whereby they damage distant organs and tissues.^16^ Systemically circulating proinflammatory cytokines stimulate receptors on other organs and tissues to release even greater numbers of cytokines.^17^ This positive feedback loop, which ultimately generates an excessive release of cytokines into the systemic circulation, is referred to as a cytokine storm.^16^ COVID‐19's cytokine storm has been the target of several treatment investigations, with recent data suggesting that IL‐6 receptor antagonists^18^ and corticosteroids^19^ improve patient survival. This cytokine storm may contribute to the hypermetabolism noted in this patient population.^6^, ^12^, ^13^, ^14^, ^15^

The heightened inflammatory state in critical illness includes a systemic inflammatory response syndrome (SIRS), defined as the presence of fever or hypothermia, tachycardia, tachypnea, and leukocytosis or leukopenia.^16^ Left unresolved, SIRS can progress to multiple organ dysfunction syndrome (MODS), typically defined as the failure of two or more organs in an acutely ill patient.^16^ Often in cases of MODS, the respiratory system is the first system to fail.^16^ The body addresses the SIRS response with a systemic compensatory antiinflammatory response syndrome, though this may also lead to complications such as immunosuppression and susceptibility to infections.^16^

In addition to inflammation, the increase in energy expenditure associated with critical illness results from neuroendocrine changes. These changes include growth hormone resistance, increased levels of glucagon and cortisol, decreased levels of insulin and testosterone, and sympathetic‐induced release of catecholamines, all of which contribute to a net catabolic state.^16^, ^20^, ^21^

The metabolic response to critical illness is characterized by an early acute phase followed by a late acute phase.^11^ The early acute phase (traditionally referred to as the “ebb” phase) lasts roughly 24–48 h and is characterized as a relatively hypometabolic state with decreased VO_2_ and decreased cardiac output.^20^ There are high‐circulating levels of glucagon and catecholamines and a high rate of endogenous energy production primarily via catecholamine‐induced glycogenolysis.^21^ After the early acute phase, the patient transitions to a late acute phase (traditionally referred to as the “flow” phase), which may last up to 7 days.^21^ The late acute phase is characterized as a hypermetabolic state with increased VO_2_ and increased cardiac output.^20^ A higher severity of illness is associated with a greater increase in energy expenditure.^20^ There is increased oxidation of nutrient stores, including increased muscle protein catabolism to synthesize acute phase protein, to yield substrate for immunoglobulins, for gluconeogenesis, and for tissue repair and synthesis.^21^

After the late acute phase, the patient transitions into either a recovery phase or a persistent inflammatory catabolic state (PICS).^11^ PICS is characterized by a prolonged state of inflammation, immunosuppression, and catabolism, ultimately leading to an increased risk for malnutrition.^16^ This may contribute to a deleterious cycle in which worsening nutrition status increases susceptibility to infection and promotes an ongoing inflammatory state.^16^ The following paragraphs summarize the results of this literature review as they relate to the metabolic changes of critical illness.

Two studies reported REE to progressively increase over the course of ICU admission in nonobese patients with COVID‐19, peaking around week 3.^14^, ^15^ Lakenman et al.^13^ did not find this progressive hypermetabolism, though limitations in study design may have precluded these inferences. Although the acute phase of critical illness is considered to last up to 1 week, this prolonged, progressive hypermetabolism may suggest there is an extended acute illness phase for severe COVID‐19. This state may relate to the relatively prolonged length of mechanical ventilation associated with COVID‐19 ARDS compared with non–COVID‐19 ARDS.^22^ One large cohort study of critically ill patients with COVID‐19 reported a median duration of mechanical ventilation of 15 days.^23^

Results were conflicting when examining this metabolic response pattern in patients with obesity. Karayiannis et al.^14^ found the same progressive hypermetabolism in patients with obesity peaking around week 3, whereas Niederer et al.^15^ essentially found that mean mREE from week 1 to weeks 2–3 did not change. Niederer et al.^15^ attributed this finding to the baseline hypermetabolic state associated with obesity (resulting from a low‐grade chronic inflammatory state), which was not significantly affected by COVID‐19, though this does not explain the disparate findings with Karayiannis et al.^14^ Patients with obesity differ from nonobese patients in their metabolic response to critical illness because of the effects of insulin resistance and relative inhibition of lipolysis, which can result in increased endogenous breakdown of lean body mass.^20^ Additional research is needed to better characterize the metabolic response pattern of patients with obesity to severe COVID‐19.

The Harris‐Benedict, WHO, and Mifflin St Jeor equations were found to underestimate energy requirements in this population.^12^, ^13^, ^15^ These equations incorporate static variables (such as height, weight, sex, and age) and, as such, do not capture other variables that affect REE, such as stress.^24^ Stress factors are commonly applied to these equations; however, the ideal stress factor to apply for critically ill patients with COVID‐19 is unknown.^24^

The case series published by Yu et al.^6^, ^12^ found patients to be in extreme hypermetabolic states. The cohort studies that followed were better designed to investigate this issue and did not consistently demonstrate these same findings^13^, ^14^, ^15^; nonetheless, the results indicate that predictive equations may significantly underestimate energy requirements for some critically ill patients with COVID‐19.

Niederer et al.^15^ found that the Penn State equations were the most accurate to predict REE in severe COVID‐19. The Penn State equations were specifically validated for ventilated patients and incorporate factors related to stress response, that is, temperature and minute ventilation.^25^ In the absence of IC, the Penn State equations have previously been suggested for use to estimate energy requirements for critically ill patients.^25^ However, predictive equations are also noted to be inaccurate in many cases, with one large retrospective cohort study of critically ill patients finding the accuracy of predictive equations to be <50%.^26^ Indeed, Niederer et al.^15^ found that the Penn State equations frequently underestimated or overestimated energy requirements.

When comparing the results of IC to clinical practice guideline recommendations for estimating energy requirements in critically ill patients (ASPEN/SCCM: 25–30 kcal/kg/day and ESPEN: 20–25 kcal/kg/day), based on the results of two studies,^14^, ^15^ the ASPEN/SCCM guidelines appear to overestimate energy requirements during the first 2 weeks of illness, whereas the ESPEN guidelines appear to underestimate energy requirements after the first 2 weeks of illness. For patients with obesity specifically, it is difficult to compare the results of these studies with clinical practice guidelines because of heterogeneity in guidelines’ recommendations for patients with obesity. ESPEN suggests estimating energy requirements for patients with obesity using adjusted body weight^11^ whereas ASPEN/SCCM suggests hypocaloric/high‐protein feeding for obese patients using either actual body weight or ideal body weight depending on obesity class.^10^ Furthermore, studies reported the results of IC in patients with obesity inconsistently (eg, adjusted body weight vs actual body weight),^14^, ^15^ limiting comparisons that can be made between the studies and with these guidelines.

The maximum study duration in this review was 7 weeks, over which time the hypermetabolic response persisted. Other acute inflammatory conditions (such as sepsis and burns) may induce hypermetabolic phases that continue for months to years, even after the initial insult or injury resolution.^27^, ^28^ In contrast, one retrospective cohort study of critically ill patients with resolved SARS‐CoV‐2 infection found patients to be normometabolic, with a mean mREE of 20 kcal/kg/day, suggesting resolution of the hypermetabolic phase after clearance of infection.^29^ In this study, repeated IC measurements were taken starting a median 17.3 days after ICU admission.^29^ As such, the duration of the hypermetabolic state with severe COVID‐19 is not well characterized.

General limitations of these studies include small sample sizes and single‐center investigations, limiting the results' generalizability. In addition, other factors that may influence REE, including sex, age, and number of comorbidities, were not described regarding differences in mREE in any studies.

## IMPLICATIONS FOR PRACTICE AND FUTURE RESEARCH

Overall, the results of this review suggest that many critically ill patients with COVID‐19 are in a hypermetabolic state, which is underestimated by predictive equations commonly used in the ICU setting. Limited published evidence suggests that the metabolic response to illness in nonobese patients is characterized by a progressive, prolonged hypermetabolic phase, peaking by week 3. The metabolic response in patients with obesity is not well characterized. The Penn State equations are the best alternatives to IC, though these equations still overestimated or underestimated mREE in many cases. As such, there is no predictive equation that can be considered a suitable alternative to IC at this time.

Of note, some groups (including ASPEN^30^ and the Australasian Society for Parenteral and Enteral Nutrition^31^) have advised against the use of IC for patients with COVID‐19 because of the increased risk of virus exposure. Other groups, including ESPEN,^32^ have suggested using IC if it can be done safely. Singer et al.^33^ have published guidance on how to reduce the risk of virus exposure while conducting IC studies.

This review highlights multiple areas for future research, including validation of the most accurate method for predicting energy requirements in critically ill patients with COVID‐19 in the absence of IC, including for nonobese patients and those with obesity; better characterization of the metabolic response to severe COVID‐19 in patients with obesity; identifying how long the hypermetabolic phase of illness lasts; and identification of any potential clinical factors or biochemical markers that are reliably associated with the hypermetabolic state. Additional considerations for research include the use of therapeutic hypothermia and neuromuscular blockade, both of which were shown to attenuate the hypermetabolic response,^12^, ^14^ as well as whether the metabolic response pattern to COVID‐19 differs based on the variant of the virus. Finally, it is suggested that authors report the results of IC in kilocalories per kilogram per day so that their findings can more readily be compared with other studies.

## CONCLUSION

Critically ill patients with COVID‐19 appear to exhibit a progressive, prolonged hypermetabolic state that is often underestimated by commonly used predictive equations in the ICU setting. Based on limited evidence published thus far, the most accurate equations for predicting energy requirements are the Penn State equations, though all predictive equations have poor individual accuracy, which increases the risk of underfeeding or overfeeding with their use. IC remains the gold standard for measuring energy expenditure in this patient population.

## CONFLICTS OF INTEREST

The authors declare no conflicts of interest.

## FUNDING INFORMATION

None declared.

## AUTHOR CONTRIBUTIONS

Ryan Burslem contributed to the conception and design of the manuscript and to the acquisition, analysis, and interpretation of the data and drafted the manusript; Kimberly Gottesman, Melanie Newkirk, and Jane Ziegler contributed to the design of the manuscript. All authors critically revised the manuscript, gave final approval, and agree to be fully accountable for ensuring the integrity and accuracy of the work.
